# Voting for Image Scoring and Assessment (VISA) - theory and application of a 2 + 1 reader algorithm to improve accuracy of imaging endpoints in clinical trials

**DOI:** 10.1186/s12880-015-0049-0

**Published:** 2015-02-19

**Authors:** Klaus Gottlieb, Fez Hussain

**Affiliations:** Quintiles, Immunology and Internal Medicine, Medical Strategy & Science, 1801 Rockville Pike Suite 300, Rockville, MD 20852 USA

**Keywords:** Central reading, Scoring methods, Randomized controlled trials, Diagnostic imaging, Clinical trial endoscopy, Outcome measurement errors, Observer variation, Binomial model, Food and Drug Administration, Inflammatory Bowel Diseases

## Abstract

Independent central reading or off-site reading of imaging endpoints is increasingly used in clinical trials. Clinician-reported outcomes, such as endoscopic disease activity scores, have been shown to be subject to bias and random error. Central reading attempts to limit bias and improve accuracy of the assessment, two factors that are critical to trial success. Whether one central reader is sufficient and how to best integrate the input of more than one central reader into one output measure, is currently not known.

In this concept paper we develop the theoretical foundations of a reading algorithm that can achieve both objectives without jeopardizing operational efficiency We examine the role of expert versus competent reader, frame scoring of imaging as a classification task, and propose a voting algorithm (VISA: Voting for Image Scoring and Assessment) as the most appropriate solution which could also be used to operationally define imaging gold standards. We propose two image readers plus an optional third reader in cases of disagreement (2 + 1) for ordinary scoring tasks. We argue that it is critical in trials with endoscopically determined endpoints to include the score determined by the site reader, at least in endoscopy clinical trials. Juries with more than 3 readers could define a reference standard that would allow a transition from measuring reader agreement to measuring reader accuracy. We support VISA by applying concepts from engineering (triple-modular redundancy) and voting theory (Condorcet’s jury theorem) and illustrate our points with examples from inflammatory bowel disease trials, specifically, the endoscopy component of the Mayo Clinic Score of ulcerative colitis disease activity. Detailed flow-diagrams (pseudo-code) are provided that can inform program design.

The VISA “2 + 1” reading algorithm, based on voting, can translate individual reader scores into a final score in a fashion that is both mathematically sound (by avoiding averaging of ordinal data) and in a manner that is consistent with the scoring task at hand (based on decisions about the presence or absence of features, a subjective classification task). While the VISA 2 + 1 algorithm is currently being used in clinical trials, empirical data of its performance have not yet been reported.

## Correspondence/Findings

### Background

This concept paper describes how greater accuracy of clinical trial outcome measures that use imaging can be achieved by using a new reading scheme that is based on rigorous theory, is operationally efficient, and can incorporate quality control. Given our therapeutic area we use the example of scoring the severity of inflammation in inflammatory bowel disease, specifically, ulcerative colitis, as example. However, we believe that VISA (Voting for Image Scoring and Assessment) can be applied more broadly to many observer-reported imaging endpoints that are based on ordinal scales.

Confirmatory phase 3 clinical trials are experiments to establish efficacy of an intervention, mostly a drug, compared to placebo or another comparator. Much attention is being focused on endpoint instruments to ensure that they are valid and accurate. Such instruments need to be able to distinguish differences between the intervention (drug) and comparator (placebo) if they indeed exist. Noisy or biased instruments will have great difficulty doing so.

It has recently been suggested that even the presumably relatively objective endoscopic subscore of the Mayo Clinic Score for ulcerative colitis disease activity, a widely used endpoint instrument for ulcerative colitis trials, is subject both to random noise and systematic bias, especially upon enrollment [1]. It seems that because investigators are eager to qualify their patients for enrollment in a trial a certain amount of upcoding of endoscopic activity scores occurs, whether this is intentional or not. Feagan et al. [1] maintained that this bias resulted in a failed trial and that scoring by an independent reader could have prevented this^a^.

Our concept paper does not examine which endoscopic scoring system is *a-priori* better, but instead investigates which reading scheme maximizes the potential of any of the available scoring systems. We argue that one independent reader is not enough, and we propose an efficient voting scheme that uses, as needed, two or three readers, including the site reader (investigator who performs the actual endoscopy). Our approach is a departure from ‘expert’-reading to jury-reading.

### Central reading, off-site reading and clinical trial endoscopy

In contrast to its equivalent in radiology or echocardiography, central reading of endoscopy is a relatively new development. The term ‘central reading’ is not well defined and perhaps is even misleading. Central reading is often understood to mean that the interpretation of imaging is not or not only done by one individual, the site reader (i.e., endoscopist at the clinical trial site), but instead is supervised, corrected, amended or adjudicated by at least one blinded off-site other reader, who as a ‘central reader’ is expected to have more expertise or less bias than the site reader. For example, as suggested by Feagan [1], site readers, may have a tendency to be biased towards more disease activity (resulting in ‘upcoding’ of the reads) to allow their patients to meet entry criteria for severity in clinical trials.

The adjective ‘central’ may come from the idea of ‘core labs’ where special expertise and advanced instrumentation historically was available. The U.S. Food and Drug Administration (FDA) uses the term off-site reading instead of central reading in one of their guidances and defines it as follows: “… offsite image evaluations are image evaluations performed at sites that have not otherwise been involved in the conduct of the study and by readers who have not had contact with patients, investigators, or other individuals involved in the study” [2].

Off-site reading is an important but not the only part of the nascent field of “Clinical Trial Endoscopy”. What investigators and trial sponsors care about are the trade-offs between accuracy, costs and operational efficiency, in other words, more than just off-site reading. In contrast to imaging in other areas, off-site reading in endoscopy introduces another dimension to the discussion. For example, no two endoscopists will record the same video of the same diseased colon. Endoscopists make choices. The variability arises, even given the same equipment, by the time spent inside the colon in general or in specific areas, the diligence with which mucus or remaining debris are washed and suctioned away, and variations in withdrawal speed, which is not constant throughout the examination. Quite literally, the site-endoscopist is in the driver’s seat and the off-site reader is a mere passenger. The site reader is an ‘embedded reporter’ and off-site readers see (to a certain extent) only what the site reader has chosen to record. This makes the contribution of the site-reader valuable, and, in our opinion, crucial. Discarding the scoring of the site reader in any reading algorithm appears wasteful if the bias of the site reader can be limited.

### Current reading schemes in clinical trial endoscopy

There is a substantial body of literature about different endoscopic scoring systems in inflammatory bowel disease [3,4]. Recently, there has been a growing interest among both researchers and regulatory agencies in how off-site reading using independent expert readers can improve performance of trials that use endoscopy as an outcome instrument [5,6]. However, there are few papers that directly address how to do this in practice (reading schemes or paradigms). These conceptual and, in our opinion, fundamental areas remain largely unexamined with the notable exception of the paper by Ahmad et al. who reviewed seven different reading schemes [7]. We believe that the word ‘paradigm’ should really only be used when referring to an accepted model, standard or prototype. The words algorithm or scheme are more neutral. While inter-observer statistics are sometimes reported, none of the reported schemes appear to be supported by a theoretical framework that attempts to justify their use or explains what implicit assumptions are being made.

### How much expertise is needed to score colonoscopy videos, a classification task?

A different way to pose this question is: Are medical experts, especially key opinion leaders in the disease area in question, able to apply the Mayo Clinic Score (or any other endoscopic outcome instrument) more accurately and more precisely than the average competent practitioner who performs endoscopy on a daily basis, sees similar patients, and was trained in the methodology?

Expert adjudication or judgment is most appropriate in instances where the knowledge domain is difficult to master and true expertise comes with decades, of experience and familiarity with rare and subtle manifestations of the entity in question.

The assignment of scores of degree of inflammation for endoscopic disease activity, while ultimately a quantification, is based on multiple relatively simple individual classification tasks that do not need this level of rare expertise: There is a finite number of elements that need to be recognized, and a typical classification task is, for example, “is this mucosal break still an erosion or already an ulcer?” Indeed, a widely-used textbook on “Measurement in Medicine” does not seem to distinguish between clinicians and experts (“Many imaging tests need a clinician or another expert to read and interpret the images”), unfortunately, the expert question is not further discussed and neither are types of classification tasks [8]. For a review of the latter see, for example, Sokolova et al. [9].

Experts seem to disagree as frequently as trainees in other endoscopic severity scoring systems such as those used for reflux esophagitis [10]. This means that the task is not difficult in the manner in which calculus, for example, is difficult (where experts would have the advantage), but that the difficulty arises from the judgment calls (i.e., subjective classifications) that need to be made.

Returning to practical applications: We had ten pairs (duos) of experts (of international or national reputation) review 30 colonoscopy videos with an approximately uniform distribution of endoscopic severity. The experts differed from each another approximately 40% of the time in the assignment of an endoscopic Mayo score (unpublished observations). These results align well with previous observations under similar circumstances [11]. The vast majority of the disagreements in our data set had a 1-step magnitude (score of 0 vs. 1, 1 vs. 2, etc., i.e., ‘boundary’ cases). In our experience, international experts do not agree amongst themselves more often than average competent and well-trained readers.

In summary, we suggest that endoscopic subscore assignment is based on simple classification tasks, and special expertise in the disease domain does not confer an advantage. Well-trained clinicians should be able to do the job. Later we will argue that voting is perhaps best suited to capture this situation numerically, as opposed to averaging, which is more appropriate for continuous measurements.

### What is the truth (gold) standard for endoscopic scoring systems?

When well-trained human readers determine a disease activity score for any given recording of a colonoscopy or other endoscopy, there is no gold standard for comparison, and accuracy can therefore not be calculated. Readers with comparable training will come to similar but slightly different scores for the same video. No individual reader, and no group of readers, can be considered ‘to set the standard’ or to be in possession of the truth. Lacking an objective evaluation outside of human readers (i.e., lacking an acceptable truth standard), there is, per definition, no criterion validity [12], and we are left with consensus-based methods [13]. In this situation, establishing construct (as opposed to criterion) validity is the next best option in determining overall validity. This requires correlation of the measurement instrument under study with other instruments that claim to measure the same construct [12].

The problem of an absent gold standard is not limited to medicine. ‘Consensus based methods^b^’ are known as knowledge aggregation in sociology, data integration in computer science, cultural consensus models in anthropology, grading a test without an answer key in psychology, Condorcet’s jury problem in political science and, less intuitively, as fault masking in reliability engineering.

Concepts can be borrowed from any of these fields but we will focus on two, fault masking by modular redundant systems, and the Condorcet jury theorem. We find those two examples attractive because they use voting algorithms which have two advantages: 1. An approach using voting corresponds to what readers do when they determine disease activity, they decide and do not measure 2. Voting algorithms avoid the calculation of means from ordinal data which most statisticians find if not unacceptable at least objectionable [14]. Once we have discussed these items and introduced our VISA reading algorithm we will revisit the truth standard issue once more.

### Why and when juries are better than single evaluators – Condorcet’s jury theorem

Condorcet’s jury theorem [15] states that a jury decision is better (in terms of the probability of being correct) than the decision of a single individual as long as the individual jurors are correct more than half the time. The more jurors, the better but with rapidly diminishing improvements as more members are added.

**Figure 1:**
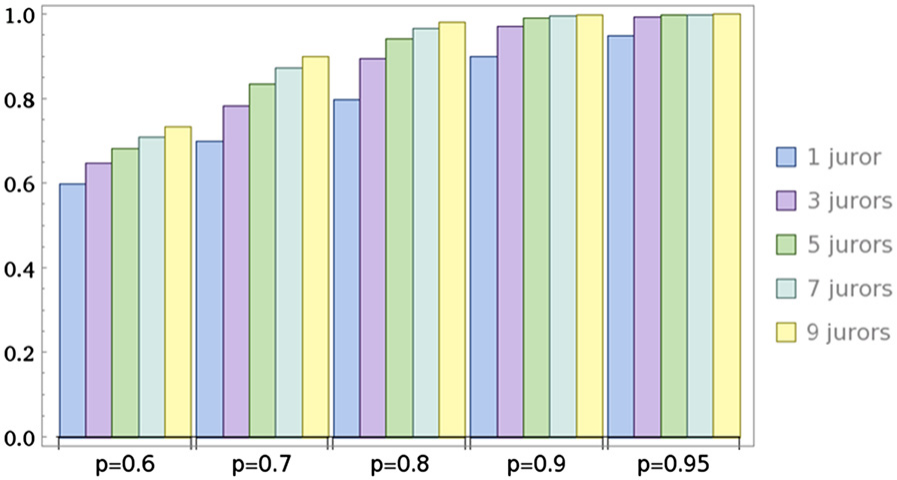
**Increasing joint probability of being correct with additional jurors.**

In Condorcet’s case a simple majority suffices for a jury to make a joint decision. This can be mathematically modeled by summing up the binomial probabilities of being correct (see Appendix for details). An uneven number of jury members prevents a tied vote. The bar graph presented as Figure 1 assumes that individually the members have probabilities between 0.60 to 0.95 of being correct (shown on the x-axis). The graph shows the calculated common probabilities on the y-axis as further jury members are added (total of 3, 5 and 7).

Inspection of this graph reveals two interesting properties. 1) The addition of a third reader, not surprisingly, leads to the steepest increase of accuracy overall. 2) The magnitude of this increase is strongest in the middle-field (single juror probabilities of being correct 0.7 - 0.8). Table 1 provides more details.

**Table 1:** **Absolute and relative gain in the joint probability (p-3 jurors) of being correct based on the individual probability (p-1 juror)**

**p-1 juror**	**p-3 jurors**	**Absolute increase in p**	**Relative increase in p**
0.6	0.648	0.048	7.4%
0.7	0.784	0.084	10.7%
0.8	0.896	0.096	10.7%
0.9	0.972	0.072	7.4%
0.95	0.993	0.043	4.3%

We can summarize that three-member juries significantly increase the probability of the jury verdict being correct if the probability of the *individual* jurors of being correct is between 0.7 and 0.8. These individual probabilities, while perhaps unachievable for juries in criminal or civil cases, can be expected from endoscopists that are well trained in the relevant scoring system. A more technical treatment of the Condorcet jury theorem can be found in the Appendix.

### Lessons from reliability engineering

**Figure 2:**
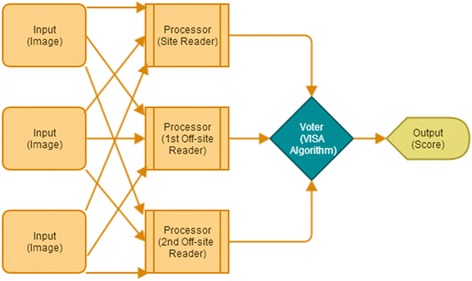
**Triple Modular Redundancy voting applied to image analysis performed by humans (adapted from Latif-Shabgahi et al.** **[** 13 **]** **.**

Reliability engineering uses software voting algorithms to increase the fault tolerance of critical systems using an approach called fault masking. One of the best known voting systems for fault masking is Triple-Modular-Redundancy: Three similar modules perform an identical function on identical input data, and then pass results to the voter. The voter arbitrates between the input results using a pre-specified strategy, and produces a single output. Different voting strategies are employed, of which the majority and median voters are commonly used [16]. It is important to note that in engineering usage the ‘voter’ is not a person but a voting schema or paradigm through which the input of the modules, in this case, the score produced by human reader, is processed. Figure 2 shows the engineering paradigm with the adaptation to imaging in parentheses (modified from reference [16]).

Some generic voting algorithms are the unanimity voter (all readers agree), the majority voter ((n + 1)/2) (which we have encountered in Condorcet’s jury theorem) and the plurality voter. In the case of three readers, majority and plurality voters are identical. More sophisticated voters, such as weighting and smoothing, and voters that consider the *a priori* probability of occurrence for each value, have been used, but their performance does not seem to be superior to the more straightforward models. In fact, it has been found that the plurality voter has the highest probability of choosing the correct result when majority, plurality, median, and mean voting algorithms are compared, and is optimal when the results of individual readers are equally likely to be correct (as assumed for Condorcet’s jury theorem) [17].

Based on the engineering solutions such as triple modular redundancy systems and our considerations of the Condorcet jury theorem, it appears that a reading scheme consisting of three modules (i.e., three independent readers) and a majority voting algorithm is the most appropriate solution.

### Are three raters always needed? If not, when are two enough?

We have already encountered triple modular redundancy. The NASA designers of the COSMOS^c^ multi-computer space borne operating system wanted to maximize accuracy (fault tolerance) while minimizing system overhead - trade-offs that are familiar to designers of clinical trials [18]. They proposed the following solution:

“… in two special cases COSMOS actually performs a 2-way vote rather than a 3-way vote. If the first two voters agree, there is no need to wait for the third to complete in order to determine the vote outcome (if the third vote turns out to be a mismatch, the mismatch will still be logged however). As an optional optimization, one of the voters may be set to”shadow” status; as a “shadow” its initiation is not even initiated unless the first two voters first finish voting and disagree with each other.”

In the following we will apply the COSMOS algorithm to reading of endoscopy scores.

### The VISA (Voting for Image Scoring and Assessment) algorithm for clinical trial imaging

**Figure 3:**
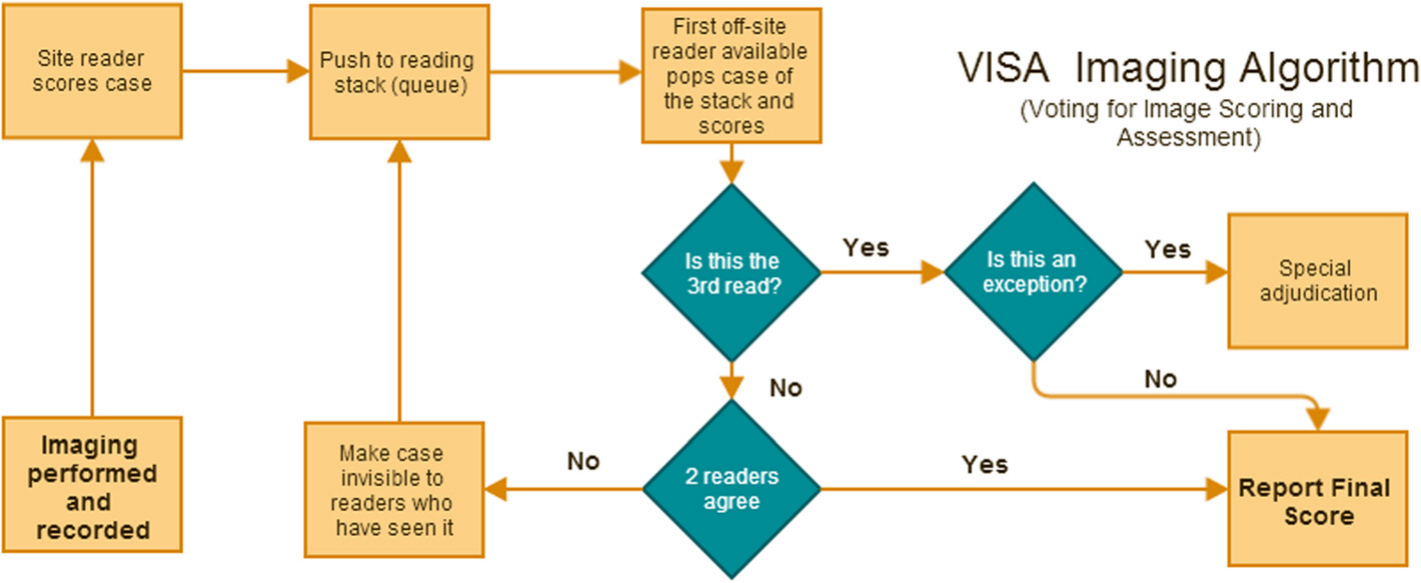
**The VISA algorithm shows how adaptive voting for image scoring (2 or 3 voters) can be efficiently implemented and automated.**

Figure 3 is a flow-diagram of the steps of the COSMOS algorithm applied to scoring of endoscopic or other imaging (renamed to VISA: Voting for Image Scoring and Assessment) that can largely be automated, i.e., implemented by software, to improve throughput. The central idea is the reading stack or queue with recycling of cases to the stack as needed (creating a logical ‘for loop’). Qualified off-site readers are notified (for example by text message) that a new case has arrived and is available for reading. The first available off-site reader logs on to the reading site and selects the case, thereby popping it off the stack and then scores the imaging case. Depending on whether the blinded off-site reader is the second or third reader, and whether there is agreement or not, different steps will be implemented as represented in the flow-chart (Figure 3).

Exceptions are anomalous or exceptional events requiring special processing that need to be defined for each specific scoring (as opposed to reading) algorithm used. For example, for the Mayo score, different readers will differ in their disease activity assessment by one step only, for example a Mayo score of 0 and 1 or 1 and 2. However, what if the final read has three different scores: 1, 2 and 3? These and other uncommon exceptions need to be defined and flagged for investigation and special adjudication. They may represent serious system breakdowns, misunderstanding and clerical errors that need to be investigated closely. If time is limited, for example, when eligibility decisions need to be made at trial entry, a decision can be made quickly by taking the median value of the three different scores while further in-depth investigations proceed in parallel.

### Revisiting the truth (gold) standard – relevance to central reading quality control

We have argued above that, lacking an objective evaluation outside of human readers, we are left with consensus-based methods for the scoring of endoscopic disease activity. These can and should be subjected to further validity testing but this will not make them gold standards. We consider quality control of the reading process to be a very important issue and so does the FDA: “We recommend evaluating reader performance with defined and prespecified metrics. Evaluation should be ongoing during the interpretation process as well as retrospective” [19].

What should these ‘defined and prespecified’ metrics be? The quoted FDA guidance is silent on the details but mentions the terms inter-reader and intra-reader variability. The choice of these terms also acknowledges the absence of acceptable gold-standards for imaging endpoints because inter-observer metrics, such as kappa, are most useful when such a standard does not exist. While these metrics have value, the limitations and the multiple pitfalls in their interpretation have been extensively discussed [17,18]. Kappa, while adjusting for chance agreement, measures concurrence and not accuracy (closeness to the truth). Kappa could be high and accuracy low if readers are all biased in the same direction, collude, or misunderstand the scoring system in a similar way. Ideally, we would like to have accuracy measures, and those require some kind of standard for comparison, i.e., a reference standard.

We hope to have shown above that it is realistic to assume that the addition of voters to a Condorcet-type jury will increase the probability of a correct voting outcome. In fact, this must be so if the individual voter’s probability of being correct does not dip below 50%, an assumption which is reasonable.

For quality control in clinical trials we could now extend our 2 + 1 algorithm to a higher number of readers, this would be more expensive and laborious, but as pointed out, even closer to the ‘truth’ than the 2 + 1 results. Since the term gold standard is suspect in this context, one could call this the “n-voter” reference standard. We think, based on the behavior of the Condorcet binomial function, that n = 5 experts would be sufficient. The results of the routine 2 + 1 voting could then be compared with the “Five-voter standard” in a sample of cases, and thus metrics beyond agreement, i.e., accuracy, could be determined. The proposed “Five-voter standard” could be further validated, in the case of endoscopic disease activity scores, for example, by establishing a correlation with histology scores.

‘Expert’ for our purposes means a person experienced in the variability of the items to be recognized and evaluated, and well trained in the specifics of the scoring system to be applied. Necessary experience would need to be defined in the individual imaging charter. In endoscopy we suggest that practitioners who are trained and credentialed to perform endoscopy in their respective jurisdictions, and currently perform endoscopies on patients with the disease for which the respective scoring system is relevant, would fulfill the minimum requirements. In addition these endoscopists would have to be trained and credentialed in the specifics of the scoring system to be used. We chose to not use the word expert *panel* because it is important that the experts evaluate the cases individually without discussing them with their peers. The results of central reading with expert panels that discuss cases amongst each another can be counter-productive [20]. In contrast, training on the scoring system should have occurred jointly. In addition to a good representation of different disease severities, there should also be a mix of easy and hard cases. Hard cases are those approaching a classification boundary or videos that are ambiguous for other reasons, for example, because the technical quality of the video is suboptimal. Unanimity is not required because this would bias against borderline cases where complete agreement cannot be expected; a simple majority suffices to formulate the reference standard.

### Quality control in practice

**Figure 4:**
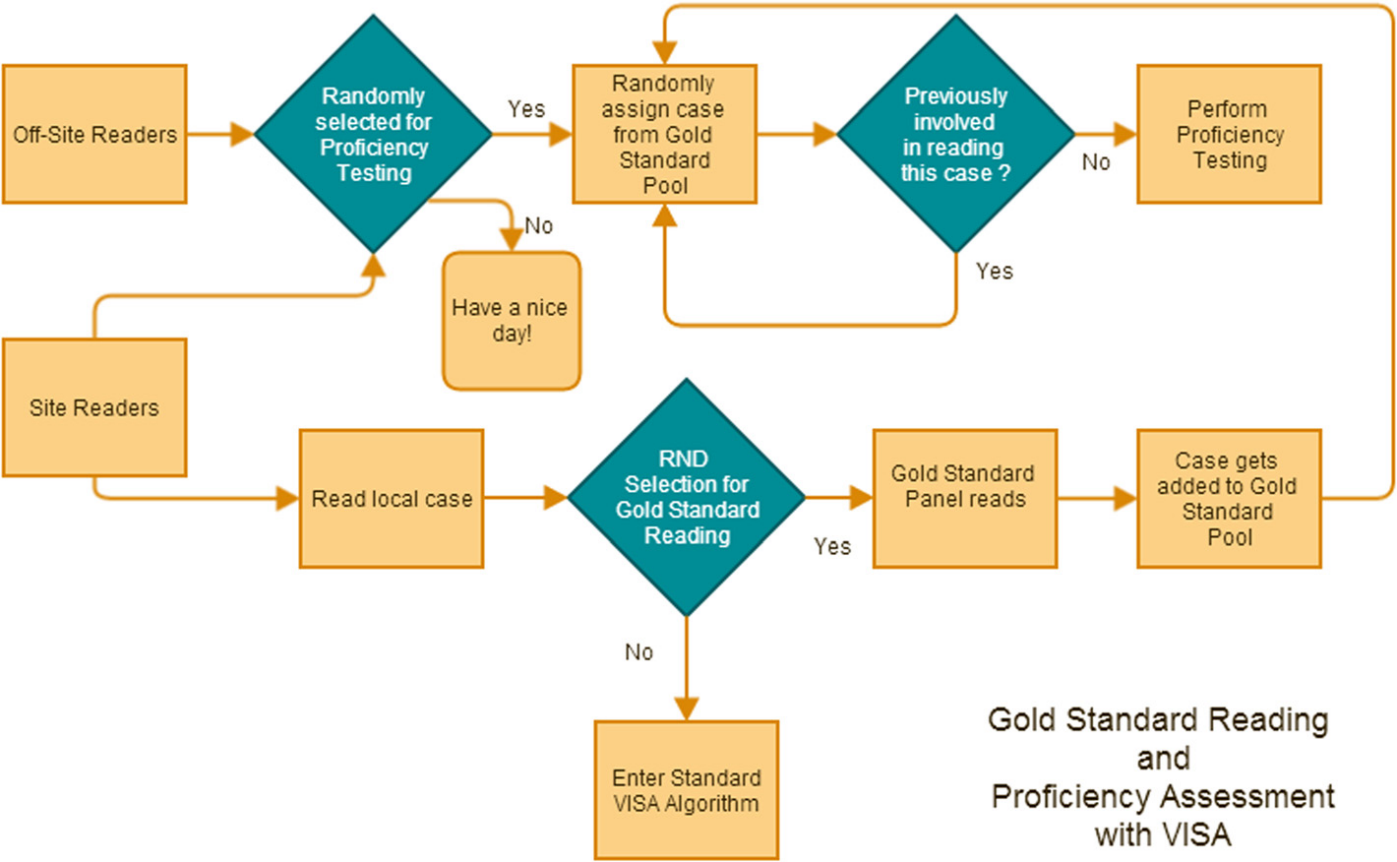
**VISA can be adjusted to create a pool of gold standard cases as the trial progresses to be used in proficiency testing.**

FDA states that as reader proficiency testing is being performed during the trial “it is important that images from the trial being assessed are not used for reader testing” [19]. That prima-vista makes sense but superior alternatives may exist. A question bank for reader testing with videos or other imaging should be relevant to the new study being conducted. First of all, the scoring of the reference cases needs to have been done with the same version of the scoring system that is relevant for the current trial. Furthermore, the test cases should reflect the types (e.g., degrees of severity) of the cases being expected in the new trial. And last, perhaps in some fast moving imaging fields the most important point, the equipment used (endoscopy, magnetic resonance imaging, positron emission tomography, etc.), should reflect the equipment that will be used in the clinical trial. All these factors make the use of preexisting test banks for reader proficiency testing during the trial less than optimal. We realize that initial reader training and qualification need to be done without the benefit of the actual clinical trial imaging files, but reader proficiency testing on random videos during the trial can be accomplished with a set of recordings set apart from the actual trial as it progresses utilizing the gold standard by voting approach (Figure 4).

## Summary and conclusion

In this concept paper we have presented a method called VISA (Voting for Image Scoring and Assessment) that promises to deliver greater accuracy of clinical trial outcome measurements that use imaging. Our theoretical framework addresses how voting improves accuracy, frames the image reading problem as a classification task, and defines the type of expertise needed to accomplish classification competently. In addition, we suggest that the number of voters can be extended from 2 + 1 (routine) to five in order to create a reference standard for quality control purposes.

We also depart from some other approaches by considering the site reader a very important part of the reading algorithm. Discarding the site reader’s contribution appears wasteful, especially in situations where image acquisition requires an expert that also knows how to score, for example, in endoscopy and echocardiography. As mentioned above, the site endoscopist performs a video recording that the subsequent reader needs to rely on. Better site reader engagement can be achieved by asking the site reader not only to record a video but also to score it while informing him or her that this score will subsequently be confirmed or challenged by off-site readers. We are confident that this will help accomplish two things: 1) Conscious up- or downcoding will decrease (i.e., scoring accuracy will increase) and 2) The video recording quality will increase allowing a better performance by the off-site readers. Knowledge of being observed (Hawthorne effect) leads to better attention to detail and it has been shown that “watching over your shoulder” increased the adenoma detection rate during colonoscopy performed for screening purposes [21]. We consider adenoma detection similar to diligent scoring and we suggest that the scoring accuracy of site readers would also increase analogous to the increase of the adenoma detection rate if site readers know that their input will be subjected to scrutiny. We think that basing a disease activity score on the judgment of a single independent off-site reader is flawed (but probably not as much as relying on the site reader alone). Instead we would like to take advantage of voting schemes that rely on a majority of the vote as the final score. This has been shown to improve accuracy above the vote (decision) of a single individual or ‘module’ (Condorcet Jury Theorem) in multiple seemingly unrelated image classification tasks. For example, automated peripheral blood smear image analyzers frequently use voting algorithms that combine the results of individual pattern classifiers [22].

A balance needs to be established between clinical, operational, scientific and cost concerns. The need for an unbiased off-site reader in IBD clinical trials has found wide currency. Reading by the site-reader should have already been included in the trial budget for the investigator grant and at least one off-site reader will be budgeted for most trials. In our implementation of VISA a software-generated automatic assignment of a third reader, if needed, is done quickly and delays in the reporting of the final score can be minimized. The question then becomes: What is the utility in terms of added cost and improved accuracy of using the VISA algorithm and adding a third reader (second off-site reader) as needed? Based on our unpublished observations mentioned above we estimate that site readers will differ from the first assigned off-site reader approximately 40% of the time (Mayo score). Different scoring systems (for example Mayo Clinic Score, Ulcerative Colitis Endoscopic Index of Severity) may perform differently but it is our opinion that overall results will be similar. We therefore estimate that a third reader will be required in 30 – 50% of reading instances. After a third read a few exceptions will occur (less than 5%). These exceptions are unique opportunities rather than a nuisance occurrence, i.e., they add value beyond the cost of the required investigations.

Altogether, we expect a 7 – 11% *relative* improvement in accuracy (see Table 1) and an approximately 30–50% cost increase for the reading process (a small portion of the overall trial cost).

Is this trade-off worthwhile? We think yes: the number of patients required for the trial could be adjusted downward, and these savings would dominate the utility analysis. Furthermore, in circumstances where the placebo rates are high and significance values closer to 0.05, increments in accuracy may well increases the chances of trial success.

The obvious limitation of our paper is the lack of concrete clinical trial evidence that shows how VISA 2 + 1 performs in comparison with other reading schemes that are currently in use. Direct head-to–head studies are unlikely. However, trials that use VISA are currently ongoing, and after the trials have been completed and publication embargoes lifted, the experience with VISA can be published. The VISA 2 + 1 read results can then easily be subjected to ‘what if analysis’, for example, asking the question, what would have happened if only one central reader would have been used (without the site reader scores)?

We hope that our paper contributes to the emerging discussion on how to best do central reading in endoscopy and we would be pleased if other imaging disciplines find it also helpful.

### Endnotes

^a^The authors of Feagan et al. do not discuss the possibility that a Hawthorne (observer) effect could have led to different results: What would site readers have done if they had known that their scores would be subjected to a second read and, based on that read, patients would have been potentially disqualified? Would they have still upcoded to the extent suspected by Feagan et al. or would their performance have been more in line with the central readers?

^b^Consensus in the broader sense; including blind assessment without communication between readers.

^c^Common Spaceborne Multicomputer Operating System and Development Environment

## Figures and Tables

**Figure 5:**
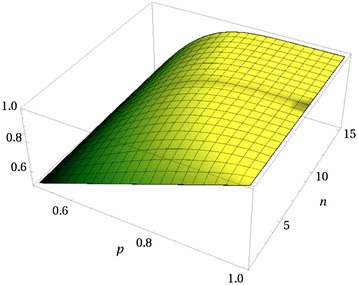
**Graph of the Condorcet equation as function of n and p.**

**Figure 6:**
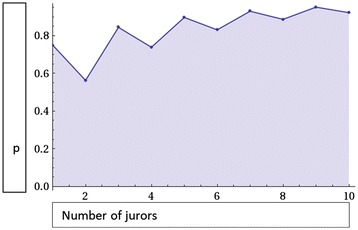
**The general case of Condorcet’s jury theorem with even and uneven numbers of jurors.**

## Appendix

### The Condorcet jury theorem

The theorem named after Marie Jean Antoine Nicolas de Caritat, Marquis de Condorcet (1743 – 1794), makes the simplifying assumption that the individual members of a jury or other decision body have equal competence (probability of making the correct choice), that this probability is greater than 0.5, and that these probabilities are independent and not correlated. It also assumes that there are only two alternatives available [23]. While these assumptions are unrealistic for modern day juries in civil or criminal cases, they are not unreasonable for bodies of experts, presumed to have sufficient training and approximately the same chance of being ‘correct’ that perform classification tasks independently without being in communication which each other. The two-alternative assumption is reasonable even in classification schemes that have more than two categories since conflicts between raters of imaging are almost always at the boundary, i.e., dichotomous. While much effort has been expended to make the Condorcet jury theorem more applicable to the political or juridical process by allowing for unequal competence and more than two choices, the ‘original’ theorem provides, in our opinion, straightforward support for the VISA voting scheme for image scoring.

The probability of the consensus being correct (P_c_) can be computed using the binomial distribution and summation of the individual probabilities for k iterations between a lower bound (k, for juries with an odd number of jurors the majority is defined by (n + 1)/2)) and an upper bound (n, the number of jurors) with p denoting the probability of being correct for individual jurors (Equation 1).

Equation 1 - Summation of binomial probabilities to arrive at the joint probability of being correct for n jurors with an individual probability of p

$$ {P}_c={\displaystyle \sum_{k=\frac{n+1}{2}}^n}\left(\begin{array}{c}\hfill n\hfill \\ {}\hfill k\hfill \end{array}\right){p}^k{\left(1-p\right)}^{n-k} $$

Readers of this paper can determine P_c_ for any arbitrary combination of n jury members (n needs to be odd, see below) and p (the individual probability of being correct) by copying the following linear notation (modifying n and p in the first two variable assignments only) into a computer algebra system or the query box of Wolfram|Alpha (http://www.wolframalpha.com/)

Equation 2 - Linear representation of equation 1 suitable for computer algebra systems

n=3 p=0.7 Sum[Binomial[n, k] p^k(1-p)^(n-k),{k,(n+1)/2,n}]

The probability of the consensus being correct (P_c_) for the example above is 0.784.

The graph of the above equation as function with two variables (n and p) for n from 1 – 15 and p from 0.51 to 1.0 is shown in Figure 5.

As can be seen, modest degrees of individual competence (expressed in p as probability of being correct for the individual) lead in the aggregate very quickly to a group competence that approaches 1 (yellow shaded area).

Equation 1 is defined for odd number of jurors because k (for majority) is defined as (n + 1)/2. Lam and Suen [24] have extended the Condorcet Jury Theorem to both odd and even numbers of readers which requires alternation between k for odd (as above) and k for even numbers of jurors (n/2 + 1) . Any number of even readers has inferior performance compared to the preceding number of odd readers, i.e., 2 readers do worse than 1 reader, 4 readers worse than 3 readers, etc.. Figure 6 shows this for p (individual) of 0.75. The preceding could be misunderstood when applied to our proposed 2 + 1 reader algorithm (VISA). In the case where a third reader is not needed (when there is agreement between the first two) the number of readers could be interpreted as even (n = 2) and, as described above, the performance should be worse than in the case of 1 reader. However, this is not so. The relevant number is how many readers *could* have participated in the vote (if needed), and that number is 3.

Readers who wish to calculate the joint probability for *even* numbers of jurors can adjust equation 2 by substituting n/2 + 1 for (n + 1)/2 in the second position between the curly brackets (parameters).
